# *N*-(2-Nitro­phen­yl)benzamide

**DOI:** 10.1107/S1600536809024271

**Published:** 2009-07-11

**Authors:** Aamer Saeed, Jim Simpson

**Affiliations:** a Department of Chemistry, Quaid-i-Azam University, Islamabad 45320, Pakistan; b Department of Chemistry, University of Otago, PO Box 56, Dunedin, New Zealand

## Abstract

In the title compound, C_13_H_10_N_2_O_3_, the central C–C(=O)–N–C amide unit makes dihedral angles of 21.68 (4) and 19.08 (4)°, respectively, with the phenyl and nitro­benzene rings. The two aromatic rings are inclined at 3.74 (3)° and the nitro group is skewed out of the attached benzene ring plane by 18.55 (8)°. An intra­molecular N—H⋯O inter­action to an O atom of the nitro substituent generates an *S*(6) ring motif. In the crystal, C—H⋯O contacts generate two centrosymmetric ring systems with *R*_2_^2^(14) and *R*_2_^2^(20) graph-set motifs, forming zigzag chains down the *a* axis. π–π inter­actions between adjacent phenyl and nitro­benzene rings [centroid–centroid distance = 3.6849 (6) Å] also form centrosymmetric dimers. These and an additional C—H⋯O hydrogen bond generate an extensive three-dimensional network structure.

## Related literature

For the biological activity of benzamide derivatives see Saeed *et al.* (2008 ▶). For related structures, see: Cronin *et al.* (2000 ▶); Glidewell *et al.* (2004 ▶); Wardell *et al.* (2005 ▶). For reference structural data, see: Allen *et al.* (1987 ▶).
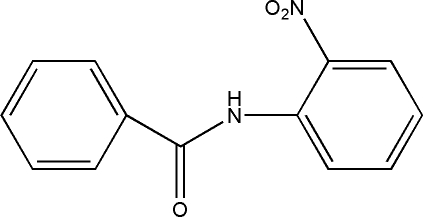


## Experimental

### 

#### Crystal data

C_13_H_10_N_2_O_3_
*M* *_r_* = 242.23
Monoclinic, 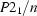

*a* = 7.2061 (5) Å
*b* = 7.4253 (5) Å
*c* = 20.6031 (13) Å
β = 93.560 (4)°
*V* = 1100.29 (13) Å^3^
*Z* = 4
Mo *K*α radiation
μ = 0.11 mm^−1^
*T* = 89 K
0.24 × 0.17 × 0.09 mm

#### Data collection

Bruker APEXII CCD area-detector diffractometer
Absorption correction: multi-scan (*SADABS*; Bruker, 2006 ▶) *T*_min_ = 0.852, *T*_max_ = 0.991
20195 measured reflections
3948 independent reflections
3098 reflections with *I* > 2σ(*I*)
*R*_int_ = 0.037

#### Refinement

*R*[*F* ^2^ > 2σ(*F* ^2^)] = 0.041
*wR*(*F* ^2^) = 0.118
*S* = 1.06
3948 reflections
167 parameters
H atoms treated by a mixture of independent and constrained refinement
Δρ_max_ = 0.49 e Å^−3^
Δρ_min_ = −0.27 e Å^−3^

### 

Data collection: *APEX2* (Bruker 2006 ▶); cell refinement: *APEX2* and *SAINT* (Bruker 2006 ▶); data reduction: *SAINT*; program(s) used to solve structure: *SIR92* (Altomare *et al.*, 1993 ▶); program(s) used to refine structure: *SHELXL97* (Sheldrick, 2008 ▶) and *TITAN2000* (Hunter & Simpson, 1999 ▶); molecular graphics: *SHELXTL* (Sheldrick, 2008 ▶) and *Mercury* (Macrae *et al.*, 2006 ▶); software used to prepare material for publication: *SHELXL97*, *enCIFer* (Allen *et al.*, 2004 ▶), *PLATON* (Spek, 2009 ▶) and *publCIF* (Westrip, 2009 ▶).

## Supplementary Material

Crystal structure: contains datablocks global, I. DOI: 10.1107/S1600536809024271/at2824sup1.cif

Structure factors: contains datablocks I. DOI: 10.1107/S1600536809024271/at2824Isup2.hkl

Additional supplementary materials:  crystallographic information; 3D view; checkCIF report

## Figures and Tables

**Table 1:** Hydrogen-bond geometry (Å, °)

*D*—H⋯*A*	*D*—H	H⋯*A*	*D*⋯*A*	*D*—H⋯*A*
N1—H1*N*⋯O3	0.887 (16)	1.927 (15)	2.6361 (11)	135.7 (13)
C10—H10⋯O2^i^	0.95	2.57	3.2254 (12)	126
C6—H6⋯O3^ii^	0.95	2.65	3.5122 (12)	151
C12—H12⋯O1^iii^	0.95	2.48	3.3695 (12)	157

Symmetry codes: (i) 
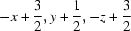
; (ii) 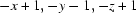; (iii) 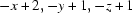.

**Fig. 1.**
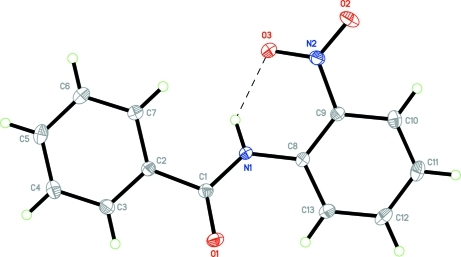
The structure of (I) with displacement ellipsoids for the non-hydrogen atoms drawn at the 50% probability level. An intramolecular hydrogen bond is drawn as a dashed line.

**Fig. 2.**
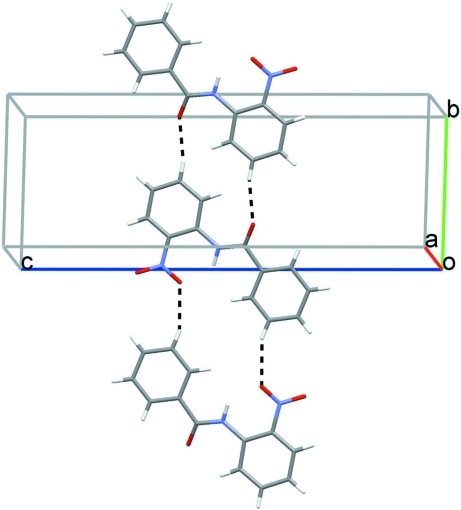
Pairs of centrosymmetric dimers forming a chain running down b axis. Hydrogen bonds are drawn as dashed lines.

**Fig. 3.**
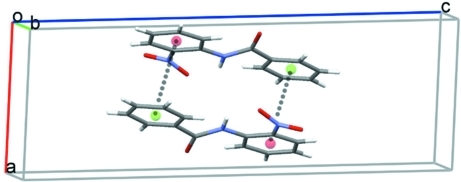
Centrosymmetric dimers formed through π–π stacking interactions shown as dotted lines with coloured circles representing the ring centroids. The symmetry operation relating the two molecules is 1 - x, -y, 1 - z.

**Fig. 4.**
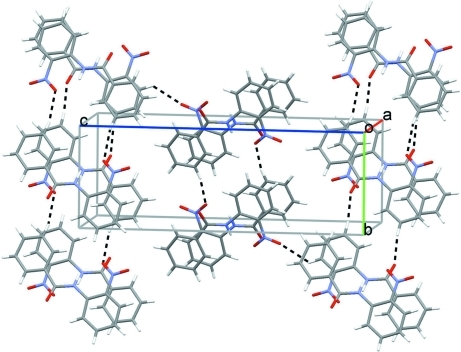
Crystal packing of (I) viewed down the a axis.

## supplementary crystallographic information

### Comment

The biological activity and applications of benzamide derivatives have been described in an earlier paper (Saeed *et al.* 2008*a*). This paper reports the structure of a nitrophenyl benzamide derivative, (I), Fig. 1. The C2–C1(O1)–N1–C8 amide unit makes dihedral angles of 21.68 (4) ° and 19.08 (4) ° with the C2–C7 and C8–C13 rings respectively. The two aromatic rings are inclined at 3.74 (3)° with the nitro group skewed out of the C8–C13 ring plane by 18.55 (8)°. An intramolecular N1—H1N···O3 interaction generates an S6 ring motif. Bond lengths in the molecule are normal (Allen *et al.* 1987) and comparable to those observed in similar structures (Cronin *et al.*, 2000; Glidewell *et al.*, 2004; Wardell *et al.*, 2005).

In the crystal C12—H12···O1 and C6—H6···O3 contacts generate two centrosymmetric ring systems with *R*_2_^2^(14) and *R*_2_^2^(20) graph set motifs respectively, forming zigzag chains down the *a* axis, Fig 2. π–π interactions between adjacent C2–C7 and C8–C13 rings [*Cg*···*Cg* distance 3.6849 (6) Å] also form centrosymmetric dimers, Fig 3. These and an additional C10—H10···O2 hydrogen bond generate an extensive three dimensional network structure, Fig. 4.

### Experimental

Freshly distilled benzoyl chloride (5.4 mmol) in CHCl_3_ was treated with 2-nitroaniline (21.6 mmol) under a nitrogen atmosphere at reflux for 3 h. Upon cooling, the reaction mixture was diluted with CHCl_3_ and washed consecutively with aq 1 *M* HCl and saturated aq NaHCO_3_. The organic layer was dried over anhydrous magnesium sulfate and concentrated under reduced pressure. Crystallization of the residue in CHCl_3_ afforded the title compound (81%) as white plates: Analysis calculated for C_13_H_10_N_2_O_3_: C 64.46, H 4.16, N 11.56%; found: C 64.39, H 4.21, N 11.71%

### Refinement

The H atom bound to N1 was located in a difference Fourier map and refined freely with an isotropic displacement parameter. The remaining aromatic H-atoms were positioned geometrically and refined using a riding model with d(C—H) = 0.95 Å, *U*_iso_=1.2*U*_eq_ (C).

### Crystal data

**Table:**

C_13_H_10_N_2_O_3_	*F*(000) = 504
*M**_r_* = 242.23	*D*_x_ = 1.462 Mg m^−^^3^
Monoclinic, *P*2_1_/*n*	Mo *K*α radiation, λ = 0.71073 Å
Hall symbol: -P 2yn	Cell parameters from 4696 reflections
*a* = 7.2061 (5) Å	θ = 2.8–31.8°
*b* = 7.4253 (5) Å	µ = 0.11 mm^−^^1^
*c* = 20.6031 (13) Å	*T* = 89 K
β = 93.560 (4)°	Plate, colourless
*V* = 1100.29 (13) Å^3^	0.24 × 0.17 × 0.09 mm
*Z* = 4	

### Data collection

**Table:**

Bruker APEXII CCD area-detector diffractometer	3948 independent reflections
Radiation source: fine-focus sealed tube	3098 reflections with *I* > 2σ(*I*)
graphite	*R*_int_ = 0.037
ω scans	θ_max_ = 33.3°, θ_min_ = 3.1°
Absorption correction: multi-scan (*SADABS*; Bruker, 2006)	*h* = −10→10
*T*_min_ = 0.852, *T*_max_ = 0.991	*k* = −11→10
20195 measured reflections	*l* = −30→30

### Refinement

**Table:**

Refinement on *F*^2^	Primary atom site location: structure-invariant direct methods
Least-squares matrix: full	Secondary atom site location: difference Fourier map
*R*[*F*^2^ > 2σ(*F*^2^)] = 0.041	Hydrogen site location: inferred from neighbouring sites
*wR*(*F*^2^) = 0.118	H atoms treated by a mixture of independent and constrained refinement
*S* = 1.06	*w* = 1/[σ^2^(*F*_o_^2^) + (0.0629*P*)^2^ + 0.2001*P*] where *P* = (*F*_o_^2^ + 2*F*_c_^2^)/3
3948 reflections	(Δ/σ)_max_ = 0.001
167 parameters	Δρ_max_ = 0.49 e Å^−^^3^
0 restraints	Δρ_min_ = −0.27 e Å^−^^3^

### Special details

**Table:**

Geometry. All e.s.d.'s (except the e.s.d. in the dihedral angle between two l.s. planes) are estimated using the full covariance matrix. The cell e.s.d.'s are taken into account individually in the estimation of e.s.d.'s in distances, angles and torsion angles; correlations between e.s.d.'s in cell parameters are only used when they are defined by crystal symmetry. An approximate (isotropic) treatment of cell e.s.d.'s is used for estimating e.s.d.'s involving l.s. planes.
Refinement. Refinement of *F*^2^ against ALL reflections. The weighted *R*-factor *wR* and goodness of fit *S* are based on *F*^2^, conventional *R*-factors *R* are based on *F*, with *F* set to zero for negative *F*^2^. The threshold expression of *F*^2^ > σ(*F*^2^) is used only for calculating *R*-factors(gt) *etc*. and is not relevant to the choice of reflections for refinement. *R*-factors based on *F*^2^ are statistically about twice as large as those based on *F*, and *R*- factors based on ALL data will be even larger.

### Fractional atomic coordinates and isotropic or equivalent isotropic displacement parameters (Å^2^)

**Table:**

	*x*	*y*	*z*	*U*_iso_*/*U*_eq_	
N1	0.73812 (11)	0.03317 (11)	0.50049 (4)	0.01315 (16)	
H1N	0.678 (2)	−0.064 (2)	0.5129 (7)	0.031 (4)*	
O1	0.87401 (11)	0.16410 (10)	0.41448 (4)	0.01879 (17)	
C1	0.78498 (13)	0.04075 (12)	0.43690 (4)	0.01259 (17)	
C2	0.71690 (13)	−0.11570 (12)	0.39614 (4)	0.01239 (17)	
C3	0.70064 (14)	−0.09052 (13)	0.32883 (5)	0.01564 (19)	
H3	0.7315	0.0226	0.3109	0.019*	
C4	0.63950 (14)	−0.23021 (14)	0.28797 (5)	0.01771 (19)	
H4	0.6275	−0.2120	0.2423	0.021*	
C5	0.59588 (14)	−0.39666 (14)	0.31405 (5)	0.0176 (2)	
H5	0.5543	−0.4921	0.2861	0.021*	
C6	0.61305 (14)	−0.42356 (13)	0.38095 (5)	0.01679 (19)	
H6	0.5840	−0.5376	0.3986	0.020*	
C7	0.67274 (13)	−0.28349 (13)	0.42198 (5)	0.01451 (18)	
H7	0.6836	−0.3018	0.4677	0.017*	
C8	0.78625 (12)	0.15306 (12)	0.55126 (4)	0.01171 (17)	
C9	0.78085 (13)	0.09917 (12)	0.61677 (4)	0.01252 (17)	
N2	0.72463 (12)	−0.08197 (11)	0.63520 (4)	0.01487 (17)	
O2	0.76511 (13)	−0.13362 (11)	0.69076 (4)	0.0268 (2)	
O3	0.63473 (11)	−0.17725 (10)	0.59477 (3)	0.01818 (16)	
C10	0.82755 (13)	0.21653 (14)	0.66792 (5)	0.01574 (19)	
H10	0.8235	0.1765	0.7116	0.019*	
C11	0.87973 (14)	0.39107 (14)	0.65503 (5)	0.0184 (2)	
H11	0.9112	0.4720	0.6897	0.022*	
C12	0.88585 (14)	0.44738 (13)	0.59089 (5)	0.01733 (19)	
H12	0.9218	0.5675	0.5820	0.021*	
C13	0.84023 (13)	0.33090 (12)	0.53965 (5)	0.01466 (18)	
H13	0.8457	0.3723	0.4962	0.018*	

### Atomic displacement parameters (Å^2^)

**Table:**

	*U*^11^	*U*^22^	*U*^33^	*U*^12^	*U*^13^	*U*^23^
N1	0.0166 (4)	0.0113 (4)	0.0116 (3)	−0.0039 (3)	0.0014 (3)	−0.0001 (3)
O1	0.0249 (4)	0.0136 (3)	0.0186 (3)	−0.0056 (3)	0.0078 (3)	−0.0007 (3)
C1	0.0126 (4)	0.0114 (4)	0.0139 (4)	0.0010 (3)	0.0017 (3)	0.0002 (3)
C2	0.0119 (4)	0.0119 (4)	0.0133 (4)	0.0005 (3)	0.0010 (3)	−0.0004 (3)
C3	0.0179 (4)	0.0153 (4)	0.0140 (4)	0.0009 (3)	0.0023 (3)	0.0007 (3)
C4	0.0190 (5)	0.0201 (5)	0.0139 (4)	0.0021 (4)	0.0000 (3)	−0.0023 (3)
C5	0.0149 (4)	0.0179 (5)	0.0198 (4)	0.0004 (3)	−0.0011 (3)	−0.0060 (4)
C6	0.0162 (4)	0.0127 (4)	0.0214 (5)	−0.0012 (3)	0.0006 (3)	−0.0013 (3)
C7	0.0154 (4)	0.0128 (4)	0.0153 (4)	−0.0001 (3)	0.0010 (3)	0.0005 (3)
C8	0.0102 (4)	0.0115 (4)	0.0135 (4)	0.0003 (3)	0.0009 (3)	−0.0008 (3)
C9	0.0126 (4)	0.0109 (4)	0.0140 (4)	0.0005 (3)	0.0009 (3)	−0.0002 (3)
N2	0.0181 (4)	0.0137 (4)	0.0130 (3)	0.0016 (3)	0.0023 (3)	0.0012 (3)
O2	0.0434 (5)	0.0226 (4)	0.0139 (3)	0.0000 (3)	−0.0016 (3)	0.0063 (3)
O3	0.0239 (4)	0.0138 (3)	0.0168 (3)	−0.0041 (3)	0.0013 (3)	0.0000 (2)
C10	0.0149 (4)	0.0179 (4)	0.0143 (4)	0.0022 (3)	−0.0001 (3)	−0.0030 (3)
C11	0.0158 (4)	0.0182 (5)	0.0212 (5)	−0.0008 (4)	0.0016 (4)	−0.0082 (4)
C12	0.0148 (4)	0.0125 (4)	0.0252 (5)	−0.0023 (3)	0.0054 (4)	−0.0038 (4)
C13	0.0145 (4)	0.0117 (4)	0.0180 (4)	−0.0009 (3)	0.0035 (3)	0.0000 (3)

### Geometric parameters (Å, °)

**Table:**

N1—C1	1.3742 (11)	C7—H7	0.9500
N1—C8	1.4006 (12)	C8—C13	1.4014 (13)
N1—H1N	0.887 (16)	C8—C9	1.4107 (13)
O1—C1	1.2250 (11)	C9—C10	1.3925 (13)
C1—C2	1.4981 (13)	C9—N2	1.4617 (12)
C2—C3	1.3971 (12)	N2—O2	1.2251 (10)
C2—C7	1.3992 (13)	N2—O3	1.2439 (11)
C3—C4	1.3902 (14)	C10—C11	1.3799 (14)
C3—H3	0.9500	C10—H10	0.9500
C4—C5	1.3915 (15)	C11—C12	1.3893 (15)
C4—H4	0.9500	C11—H11	0.9500
C5—C6	1.3906 (14)	C12—C13	1.3883 (13)
C5—H5	0.9500	C12—H12	0.9500
C6—C7	1.3914 (13)	C13—H13	0.9500
C6—H6	0.9500		
			
C1—N1—C8	128.45 (8)	C2—C7—H7	119.9
C1—N1—H1N	117.4 (10)	N1—C8—C13	122.01 (8)
C8—N1—H1N	113.9 (10)	N1—C8—C9	120.93 (8)
O1—C1—N1	123.75 (9)	C13—C8—C9	117.06 (8)
O1—C1—C2	121.96 (8)	C10—C9—C8	121.79 (9)
N1—C1—C2	114.29 (8)	C10—C9—N2	115.92 (8)
C3—C2—C7	119.32 (9)	C8—C9—N2	122.29 (8)
C3—C2—C1	117.20 (8)	O2—N2—O3	122.11 (9)
C7—C2—C1	123.47 (8)	O2—N2—C9	118.49 (8)
C4—C3—C2	120.34 (9)	O3—N2—C9	119.38 (8)
C4—C3—H3	119.8	C11—C10—C9	119.87 (9)
C2—C3—H3	119.8	C11—C10—H10	120.1
C3—C4—C5	119.97 (9)	C9—C10—H10	120.1
C3—C4—H4	120.0	C10—C11—C12	119.39 (9)
C5—C4—H4	120.0	C10—C11—H11	120.3
C6—C5—C4	120.13 (9)	C12—C11—H11	120.3
C6—C5—H5	119.9	C13—C12—C11	121.05 (9)
C4—C5—H5	119.9	C13—C12—H12	119.5
C5—C6—C7	119.99 (9)	C11—C12—H12	119.5
C5—C6—H6	120.0	C12—C13—C8	120.83 (9)
C7—C6—H6	120.0	C12—C13—H13	119.6
C6—C7—C2	120.23 (9)	C8—C13—H13	119.6
C6—C7—H7	119.9		

### Hydrogen-bond geometry (Å, °)

**Table:**

*D*—H···*A*	*D*—H	H···*A*	*D*···*A*	*D*—H···*A*
N1—H1N···O3	0.887 (16)	1.927 (15)	2.6361 (11)	135.7 (13)
C10—H10···O2^i^	0.95	2.57	3.2254 (12)	126
C6—H6···O3^ii^	0.95	2.65	3.5122 (12)	151
C12—H12···O1^iii^	0.95	2.48	3.3695 (12)	157

Symmetry codes: (i) −*x*+3/2, *y*+1/2, −*z*+3/2; (ii) −*x*+1, −*y*−1, −*z*+1; (iii) −*x*+2, −*y*+1, −*z*+1.
